# Functional Outcome of Varus Derotation Osteotomy in Legg-Calve-Perthes Disease: Can It Be Justified in Late-Presenting Disease?

**DOI:** 10.7759/cureus.49788

**Published:** 2023-12-01

**Authors:** Jaya V Lal, Sagar Tontanahal, Joseph Francis, Kevin M Philip, Ramesh LJ, Binu T Kurian

**Affiliations:** 1 Orthopaedics, St. John's Medical College Hospital, Bangalore, IND; 2 Orthopaedics, Dr. Chandramma Dayananda Sagar Institute of Medical Education and Research, Bangalore, IND

**Keywords:** paediatric outcomes data collection instrument, modified elizabeth town classification, late presenting perthes, varus derotational osteotomy, legg-calvé-perthes disease

## Abstract

Background: Legg-Calve-Perthes disease (LCPD) in children older than seven years has often been associated with accelerated progress and poor outcome. The results of varus derotation osteotomy (VDRO) of the proximal femur in this cohort are not consistently predictable. This study was aimed at assessing the functional outcome of VDRO for hip containment in children with late-presenting LCPD.

Materials and methods: A quasi-prospective observational study was conducted to determine the functional outcomes of children with late-presenting unilateral LCPD who underwent VDRO between 2016 and 2021, with a minimum follow-up of two years. A retrospective chart review followed by a patient/parent-reported outcome measure using the Paediatric Outcome Data Collection Instrument (PODCI) was utilised.

Results: Thirteen children were included in this study, with a mean age of 8.30 years (range: 7-12 years; SD: -1.493). Three children were in the early stages of the disease, modified Elizabethtown I and IIA (1 and 2, respectively). The majority of the children were in Stage IIB of the modified Elizabethtown staging (n=6), followed by Stage IIIA (n=4). The two children presenting in Stage IV of the disease were excluded from the analysis. The mean standardised and normative PODCI scores for transfer and mobility were 98.23 and 48.03, respectively. The mean standardised and normative PODCI scores for sports and physical were 93.15 and 49.76, respectively. Neither of the scores showed a statistically significant difference between the late and early stages of the disease (Transfer and Basic Mobility Scale: Standardised (p=0.273), Normative (p=0.268); Sports and Physical Functioning Scale: Standardised (p=0.618), Normative (p=0.631)). However, a higher mean PODCI score was noted for the early stages. There was no statistically significant difference between the median score and the duration since surgery. However, there was a moderate negative correlation between the time scores and the times since surgery for the late stages of the disease, viz. Stage IIB and IIIA (Transfer and Basic Mobility Scale: Standardised (-0.445), Normative (-0.450); Sports and Physical Functioning Scale: Standardised (-0.228), Normative (-0.228)). This correlation, however, did not reach a statistical significance.

Conclusion: VDRO can be regarded as a functionally rewarding option for femoral head containment in late-presenting LCPD across the evolutionary stages of the disease.

## Introduction

Legg-Calve-Perthes disease (LCPD) is a self-limiting disorder of the paediatric hip distinguished by aseptic necrosis of the femoral head, subchondral fracture, fragmentation, revascularization, and remodelling [1,2]. The disease is considered as late-presenting when the symptomatology begins after seven years of age [3]. The age of the patient at diagnosis is a significant prognostic factor; children older than seven years have a worse prognosis than younger children [4-6]. 

All therapeutic strategies aim at containing the femoral head in the acetabulum to prevent femoral head deformity and preserve joint congruity. These can be done by surgical or non-surgical means, and procedures performed prior to the stage of revascularization have the best results [7,8].

In spite of the availability of various treatment options, there is still debate over the most appropriate treatment for children with late-presenting LCPD, with literature showing conflicting conclusions. Our study aimed to assess the functional outcome of children with late-presenting LCPD treated with varus derotation osteotomy (VDRO).

## Materials and methods

We conducted a quasi-prospective observational analysis of patients with late-presenting LCPD who underwent VDRO at our institution between January 2016 and January 2021. Hospital records of these patients were accessed after obtaining approval from the Institutional Ethics Committee at St. John's Medical College Hospital, Bangalore (Approval No.: 111/2023, approved on May 5, 2023). Children diagnosed with unilateral LCPD who underwent VDRO and had a minimum follow-up of two years were included in the study. Children with healed disease, bilateral disease, who underwent other forms of containment, or had inadequate follow-up were excluded from the study. Of the 28 cases of LCPD treated with VDRO in our institution, 13 met the inclusion criteria and were selected for analysis. 

Children were assessed preoperatively with radiographs, and the stage of the disease was classified according to the modified Elizabethtown classification [9]. Regular radiographic and clinical follow-up was done, and weight-bearing was allowed for the children following the union of the osteotomy. A single observer administered the Paediatric Outcome Data Collection Instrument (PODCI) via telephone, a minimum of two years following the surgery.

PODCI is a patient-reported outcome measure (PROM) originally developed in 1994 as the Paediatric Orthopedic Association of North America outcomes instrument [10]. The PODCI initially emerged to evaluate changes in outcome following surgical intervention in various musculoskeletal diseases [11-13]. Being a PROM, PODCI provides real-time, patient-centric data that can be used to assess the efficacy of therapeutic interventions [14,15].

Surgical technique

The surgical procedure was carried out by the same senior orthopaedic surgeon. The proximal femur was exposed using the standard lateral subvastus approach with the child in supine position. A lateral-based open wedge osteotomy was performed at the subtrochanteric level to achieve a varus and internal rotation of 15°-20°. The osteotomy site was secured with a pre-bent 3.5 mm dynamic compression plate and screws. Intraoperative fluoroscopic assessment of femoral head containment was performed.

Rehabilitation protocol

Following surgery, hip spica casts were applied for at least six weeks, following which they began a progressive hip range of motion exercise regimen and guarded weight-bearing until the osteotomy site had completely healed. By the completion of this review, hardware removal was performed for three out of 13 patients.

Statistical analysis

We used IBM SPSS Statistics for Windows, Version 20.0 (Released 2011; IBM Corp., Armonk, NY, United States) for the analysis of our results. ANOVA test was used to assess the correlation between the PODCI score and the stage of the disease. A non-parametric test was performed to assess the correlation between the PODCI score and the time duration between surgery and administering the PODCI score. The statistical significance value (p) was set to 0.05.

## Results

Thirteen children who met the inclusion criteria and were available for retrospective follow-up were analysed. The mean age at presentation was 8.30 years (range: 7-13 years, SD: 1.493), with a male preponderance (7/13, 53%). In our cohort, the majority of the cases belonged to Stage IIB and Stage IIIA of the modified Elizabethtown classification (6, 46% and 4, 30%, respectively) (Table 1).

**Table 1:** Descriptive statistics. PODCI: Paediatric Outcome Data Collection Instrument.

Parameter	Frequency
Mean age (in years)	8.30±1.49
Gender (M:F)	07:06
Laterality (R:L)	08:05
Modified Elizabethtown Classification	
Stage IA	0
Stage IB	1 (7.6%)
Stage IIA	2 (15.3%)
Stage IIB	6 (46.1%)
Stage IIIA	4 (30.7%)
Adolescent mean PODCI score	
Transfer and Basic Mobility Scale: Standardised mean (0 to 100)	98.23±3.13
Transfer and Basic Mobility Scale: Normative score (-164 to 52)	48.07±6.92
Sports and Physical Functioning Scale: Standardised mean (0 to 100)	93.15±8.28
Sports and Physical Functioning Scale: Normative score (-36 to 56)	49.76±7.55
Residual complaints at two years	
Limp	7
Pain while walking	0
Limb length discrepancy	0
Fixed hip deformity	0
Restriction of movements	4
Ambulating with assistance	0
Infection	0

The transfer and basic mobility and sports and physical functioning subcomponents of the PODCI score were administered to each patient. ANOVA test was used to assess the correlation between the PODCI score subcomponents and modified Elizabethtown Stage of the disease. Neither of the scores showed a statistically significant difference across the stages of the disease (Transfer and Basic Mobility Scale: Standardised (p=0.620), Normative (p=0.629); Sports and Physical Functioning Scale: Standardised (p=0.283), Normative (p=0.287)).

Pearson correlation coefficient was used to analyse the correlation of the PODCI score with the time duration between the surgical intervention and administering the PODCI score. There was no statistically significant difference between the median PODCI score and the duration since surgery. There was, however, a moderate negative correlation between the time scores and the time since surgery for the late stages of the disease, namely Stage IIB and IIIA (Transfer and Basic Mobility Scale: Standardised (-0.445), Normative (-0.450); Sports and Physical Functioning Scale: Standardised (-0.228), Normative (-0.228)). This correlation, however, did not reach a statistically significant difference (Table 2).

**Table 2:** Comparison of PODCI score with stage of disease and duration since surgery. PODCI: Paediatric Outcomes Data Collection Instrument.

Adolescent PODCI score component (mean)	Modified Elizabethtown stage (p-value)	Time duration between surgery and PODCI score administered (Pearson correlation coefficient)
Transfer and Basic Mobility Scale: Standardised mean	0.620	-0.445
Transfer and Basic Mobility Scale: Normative score	0.629	-0.450
Sports and Physical Functioning Scale: Standardised mean	0.283	-0.228
Sports and Physical Functioning Scale: Normative score	0.287	-0.228

Independent Samples t-test was used to assess the correlation between PODCI scores in the early and late stages of the disease (Early stages: IA, IB, and IIA; Late stages: IIB, IIIA, IIIB, and IV). Neither of the scores showed a statistically significant difference between the late and early stages of the disease (Transfer and Basic Mobility Scale: Standardised (p=0.273), Normative (p=0.268); Sports and Physical Functioning Scale: Standardised (p=0.618), Normative (p=0.631)). However, a higher mean PODCI score was noted for the early stages (Table 3).

**Table 3:** Correlation of adolescent PODCI score with the early and late stages of LCPD. PODCI: Paediatric Outcomes Data Collection Instrument; LCPD: Legg-Calve-Perthes disease.

Adolescent PODCI score component	Early stages	Late stages	p-value
	(Mean score)	(Mean score)	
Transfer and Basic Mobility Scale: Standardised mean	100	97.8	0.273
Transfer and Basic Mobility Scale: Normative score	52	47.2	0.268
Sports and Physical Functioning Scale: Standardised mean	95.3	92.6	0.618
Sports and Physical Functioning Scale: Normative score	51.7	49.3	0.631

**Figure 1:**
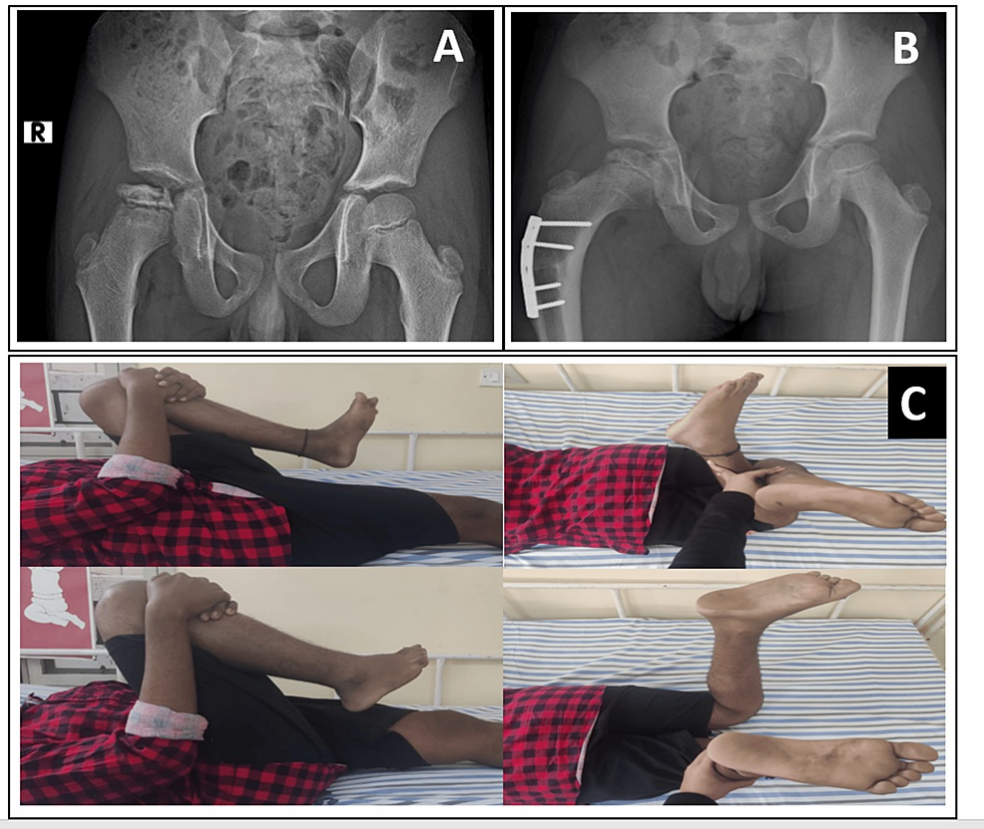
A 10-year-old boy with LCPD. (A) Preoperative radiograph showing modified Elizabethtown Stage IIB. (B) Postoperative radiograph taken at a follow-up of two years. (C) Range of movement found satisfactory at a follow-up of two years. LCPD: Legg-Calve-Perthes Disease.

**Figure 2:**
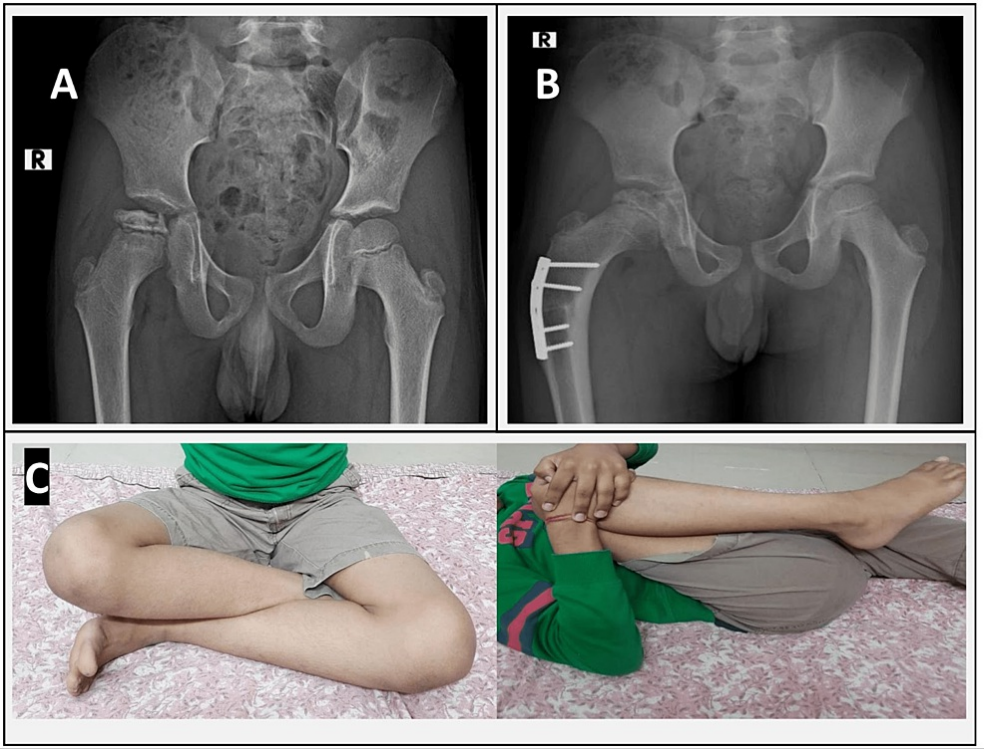
An eight-year-old boy with LCPD. (A) Preoperative radiograph showing modified Elizabethtown Stage IIB. (B) Postoperative radiograph at a follow-up of two years. (C) Range of movement found satisfactory at a follow-up of two years. LCPD: Legg-Calve-Perthes Disease.

The most common postoperative complication reported by the parents and children was a limp (6/13; 46%). None of the children had any fixed deformities, but four children reported terminal restriction of abduction and internal rotation, which however did not affect their normal daily activities. There were no complaints of persistent hip pain on ambulation or infections seen in the study (Figures 1, 2).

## Discussion

In spite of over a century of dedicated research, controversy still exists regarding the most effective treatment for late-presenting LCPD. The cohort in this study has a mean age of 8.30 years (range: 7-13 years; SD: 1.493). The age distribution is similar to the studies that have analysed the outcome following containment surgery in this subset of the disease (Table 4) [16-18]. Age at presentation is a crucial factor in determining the outcome of LCPD in children, with substantial data showing young children with the disease do significantly better in the long run [19,20]. However, it is unclear if this finding can be extrapolated to a subset of late-presenting LCPD. In this study, neither of the PODCI scores showed a statistically significant difference with the age of the child (Transfer and Basic Mobility Scale: Standardised (p=0.511), Normative (p=0.524); Sports and Physical Functioning Scale: Standardised (p=0.833), Normative (p=0.794)).

**Table 4:** Characteristics of studies with similar age cohort. HHS: Harris hip score; EQ: epiphyseal quotient.

Study	Method	Participants	Mean age (range)	Patient distribution		Outcome	
Joshi et al. [16]	Prospective	15	9.4 years (8-12)	Modified Elizabethtown		HHS: 93.2 (86-100)	
				Stage IB	5	EQ	
				Stage IIA	6	Good	9
				Stage IIB	4	Fair	4
						Poor	2
Noonan et al. [17]	Retrospective	17	10.6 years (9.5-13.9)	Catterall group		Mose criteria	
				2	1	Good	4
				3	12	Fair	2
				4	5	Poor	11
				Lateral pillar classification		Stuhlberg criteria	
				A	4	1	2
				B	8	2	4
				C	6	3	4
						4	5
						5	2
Stepanovich et al. [18]	Retrospective	54	8.2 years (6.4-10)	Modified Elizabethtown		Stuhlberg criteria	
						I/II	III/IV
				Early (I, IIA)	27	17	10
				Late (>IIB)	29	19	10
				Catterall group			
				I/II	10	8	2
				III/IV	46	28	18
				Lateral pillar classification			
				B and BC	39	28	11
				C	17	8	9

The favourable role of containment surgery in older children is also well documented in the literature [17,21,22]. When performed during the early stages of the disease, until Stage IIA of the Modified Elizabethtown Classification, the chances of achieving a spherical head with a reasonably well-preserved range of hip motion are high [16,22]. This trend was also seen in our study, with children undergoing VDRO in the early stages of the disease having a greater range of motion and higher functional scores, as the mean PODCI score for children in the early stages was higher than that in the late stages (Table 3).

An interesting finding in our study was that children who operated later in the course of the disease, i.e., in Stage IIB and IIIA, reported good functional scores. Although the scores were lower than the children in the early stages, the difference did not reach statistical significance (Transfer and Basic Mobility Scale: Standardised (p=0.273), Normative (p=0.268); Sports and Physical Functioning Scale: Standardised (p=0.618), Normative (p=0.631)). This observation was supported by a study by Stepanovich et al. [17]. This could indicate a possible benefit of containment surgery in the functionality of these children, which to this date remains a point of contention in LCPD.

Furthermore, the trends of good functional outcomes have been seen even for longer durations following surgery, a mean of four years since surgery, indicating that the improvement is not a transient effect. We, however, do have our reservations about saying that children in later stages of disease will continue to function well into adulthood because functional scores do show a decline with time since surgery.

The study's sample size and fairly limited duration of follow-up were a few limitations that must be acknowledged. However, this study does show a positive effect of VDRO in the late stages of disease, a concept not widely advocated. A larger long-term follow-up study to analyse how these hips fare in adulthood would be ideal to identify the optimal management protocol for LCPD.

## Conclusions

Treatment of late-presenting LCPD with VDRO of the proximal femur exhibits satisfactory functional outcomes across the evolutionary stages of the disease. Further, large-scale prospective trials with long-term follow-up are vital to assess the true benefits and restrictions of VDRO at various stages of LCPD evolution and how these results translate into adulthood.
